# Adaptive Multi-Camera Fusion and Calibration for Large-Scale Multi-Vehicle Cooperative Simulation Scenarios

**DOI:** 10.3390/s26030977

**Published:** 2026-02-03

**Authors:** Hui Zhang, Chenyu Xia, Huantao Zeng

**Affiliations:** 1 School of Intelligent Systems Engineering, Sun Yat-sen University, Shenzhen 518107, China; zhanghui@mail.sysu.edu.cn (H.Z.); xiachy@mail2.sysu.edu.cn (C.X.); 2 China Telecom Co., Ltd., Guangdong Branch, Guangzhou 510030, China

**Keywords:** image stitching, multi-camera vision, real-time processing

## Abstract

In the development of multi-vehicle cooperative hardware-in-the-loop (HIL) simulation platforms based on machine vision, accurate vehicle pose estimation is crucial for achieving efficient cooperative control. However, monocular vision systems inevitably suffer from limited fields of view and insufficient image resolution during target detection, making it difficult to meet the requirements of large-scale, multi-target real-time perception. To address these challenges, this paper proposes an engineering-oriented multi-camera cooperative vision detection method, designed to maximize processing efficiency and real-time performance while maintaining detection accuracy. The proposed approach first projects the imaging results from multiple cameras onto a unified physical plane. By precomputing and caching the image stitching parameters, the method enables fast and parallelized image mosaicking. Experimental results demonstrate that, under typical vehicle speeds and driving angles, the stitched images achieve a 93.41% identification code recognition rate and a 99.08% recognition accuracy. Moreover, with high-resolution image (1440 × 960) inputs, the system can stably output 30 frames per second of stitched image streams, fully satisfying the dual requirements of detection precision and real-time processing for engineering applications.

## 1. Introduction

Multi-vehicle cooperation, as an advanced intelligent vehicle control strategy, can effectively improve traffic efficiency and ensure transportation safety. For algorithm validation, three primary approaches are commonly used: pure simulation, physical experiments, and hardware-in-the-loop (HIL) or semi-physical simulation. Among these, video-based semi-physical simulation offers notable advantages in terms of cost efficiency and high accuracy.

The semi-physical simulation platform developed in this study employs an overhead vision system to detect and identify the identity, position, velocity, and heading angle of model vehicles within a simulated road network. The model vehicles execute control commands solely based on wireless instructions for speed and steering, while the perception and decision-making functions required for simulating intelligent vehicles are implemented through the backend server software. This architecture not only adapts flexibly to various multi-vehicle cooperation scenarios but also eliminates the complexity of model vehicle integration and debugging, providing a safe and efficient environment for cooperative algorithm testing.

The core challenge in constructing such a system lies in the detection and recognition of vehicle information from overhead video within a simulated large-scale road network. This primarily involves two technical problems: multi-camera image fusion in large-scale environments and vehicle state recognition. Among these, the former is the focus of this paper, and its main difficulties include:

(1) the absence of standard reference objects for calibration (as the simulated road network may change dynamically).
(2) potential rotational misalignment among individual camera coordinate systems over time (due to installation shifts or mechanical loosening).

To address these challenges, this work proposes an adaptive calibration and fusion algorithm. The method utilizes known identity codes and road markings (such as lane lines) as reference features. By analyzing the size, shape, and positional variation of identity codes on moving model vehicles, the system estimates the necessary correction parameters for multi-camera alignment. Since a single correction may be insufficient, the calibration process is performed iteratively—the system updates correction parameters, re-controls the vehicle motion, and repeats the procedure until two consecutive correction values converge.

The effectiveness of this approach can be validated by evaluating the positional error between actual and detected coordinates, as well as by assessing the continuity of vehicle trajectories during motion.

Driven by the development needs of a semi-physical simulation system, this study aims to construct a high-performance multi-vehicle cooperative simulation platform and verify its effectiveness. A “hardware–algorithm–multi-scenario validation” research framework is established, with the following objectives:

(1) to build an efficient simulation platform for multi-vehicle cooperation.
(2) to develop spatiotemporal trajectory planning examples for platform evaluation.
(3) to validate and assess the overall performance and reliability of the proposed simulation platform.

**Figure 1:**
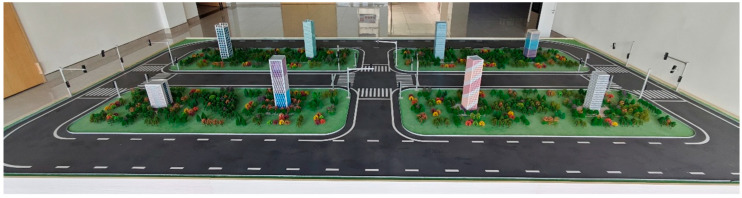
Photograph of the Simulation System.

**Figure 2:**
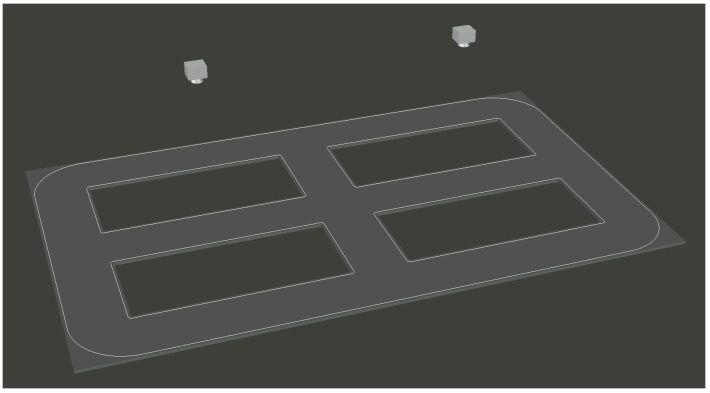
Schematic Diagram of the Camera Installation Setup (Two Cameras in the Figure).

During the development of the simulation platform, as illustrated in Figure 1, the system adopts a camera-based approach to capture the entire scene and identify vehicle identities within it. The current camera installation configuration, shown in Figure 2, employs undistorted 120° wide-angle cameras to cover the entire area. However, the peripheral regions of the captured scene often exhibit reduced recognition performance due to lens distortion and resolution attenuation. Therefore, enhancing video clarity to improve vehicle recognition accuracy becomes a crucial issue in this study, for which video image stitching provides an effective solution.

Images captured from multiple cameras often suffer from lens distortion and viewpoint discrepancies. A critical research challenge lies in correcting and aligning these images within a unified coordinate system, ensuring that no visible seams or distortions occur during stitching. Existing studies primarily adopt camera calibration-based correction methods [1,2,3,4], which achieve geometric rectification and perspective alignment by acquiring both the intrinsic parameters (such as focal length, principal point, and distortion coefficients) and the extrinsic parameters (position and orientation) of the cameras.

In recent years, driven by the advancement of autonomous driving perception technologies, several studies have introduced Bird’s-Eye View (BEV)-based feature projection calibration methods [5,6,7], such as CalibRBEV, which can perform extrinsic optimization and coordinate alignment among multiple cameras without requiring physical calibration targets. These methods significantly enhance engineering deployability and adaptive capability in dynamic environments.

In multi-view image composition tasks, image stitching technology [8,9] plays a critical role, as the quality of the stitched image directly affects the reliability of input data for subsequent object detection and recognition modules. The stitching process typically involves three key stages: feature extraction and matching, image registration, and seam optimization. Among them, image registration—which determines the geometric transformation relationships among multiple images to achieve accurate alignment within a common coordinate frame—is the core step that most strongly influences stitching precision.

Depending on the criteria used for registration, existing methods [10,11,12] can be broadly classified into region-based and feature-based approaches.

Region-based methods [13,14,15] directly use pixel intensity or color distribution information to perform image matching via similarity metrics such as cross-correlation or mutual information. These approaches are theoretically well-founded and perform well in texture-rich scenes with limited geometric variation, but they lack robustness under conditions of large-scale changes, strong rotations, or weak texture patterns.

Feature-based methods [16,17,18], in contrast, first extract key feature points (e.g., SIFT—Scale-Invariant Feature Transform, and SURF—Speeded-Up Robust Features) and then compute the transformation matrix through feature point correspondence. These methods exhibit greater stability and adaptability to scale variation, rotation, occlusion, and illumination changes, making them widely used in complex environmental image stitching tasks.

To further improve the global consistency and visual quality of stitched images, recent research has introduced deep learning-based stitching algorithms [19,20,21], such as UDIS (Unsupervised Deep Image Stitching), which leverage convolutional neural networks (CNNs) to extract deep multi-view features and achieve content completion and seam refinement in non-overlapping regions. Although these deep stitching models offer superior performance in complex scenarios, their computational complexity often limits real-time applicability. Therefore, in engineering-oriented applications, a balance must be struck between visual quality and computational efficiency.

In this study, focusing on the video recognition module within a semi-physical simulation system, we propose an image stitching algorithm designed for low-cost, high-efficiency target detection in practical engineering contexts. Unlike most existing studies that emphasize only the visual quality of the stitched result, the proposed method integrates image correction and direct stitching to achieve real-time performance while maintaining adequate recognition accuracy for large-scale multi-vehicle cooperative simulations.

## 2. Materials and Methods

### 2.1. Background and Overall Design of the Fast Image Stitching Algorithm

**Figure 3:**
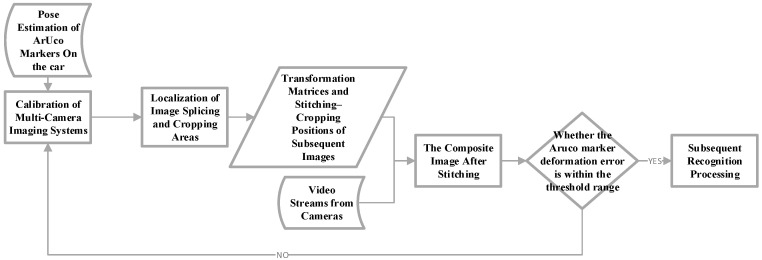
Overall Architecture of the Proposed Method.

The overall workflow of the method proposed in this article, which stitches two or more video streams into a single continuous video stream, is illustrated in Figure 3. In this design, video images from multiple cameras are first rectified multiple times using the ID markers mounted on the vehicle moving within the scene. This process maps the images onto a unified reference plane, thereby eliminating geometric discrepancies caused by differences in camera angles and other factors.

Subsequently, when the vehicle enters the overlapping region of the cameras, the positions of the ID markers are identified within the rectified images. Based on these positions, the stitching regions and parameters for the multiple video streams are pre-calculated, enabling efficient video mosaicking in subsequent processing.

Compared with traditional stitching strategies that perform feature point extraction and matching on every frame, the proposed video stitching method offers significant efficiency advantages. Since the stitching regions and parameters are determined during the system’s initialization stage, it is unnecessary to repeatedly execute complex feature extraction and matching computations for each incoming video frame in subsequent processing. This design greatly reduces the computational complexity of the system and provides robust technical support for real-time, high-efficiency engineering applications.

### 2.2. Algorithm Implementation

For target detection, two main approaches are commonly used: frame-based comparison methods, such as the frame-difference method, and feature-based detection methods. OpenCV, as a powerful open-source computer vision library, provides extensive functionality that greatly facilitates algorithm implementation. Among its components, the Aruco marker library, introduced in OpenCV 4, offers highly robust capabilities, including the identification of marker IDs and the estimation of each marker’s position and orientation.

In this design, a feature-based recognition approach is adopted, where Aruco markers with different IDs are assigned to different vehicles as unique features. This enables fast and accurate recognition of each vehicle’s pose and identity within the scene.

Furthermore, the approach of stitching multiple low-resolution camera images eliminates the need for expensive high-resolution cameras. Additionally, the proposed method avoids employing highly complex algorithms, enabling it to excel at video stitching on the experimental machine (Intel i7, RTX 2060) without requiring excessive computational power.

#### 2.2.1. Adaptive Image Calibration

The purpose of image rectification is to eliminate errors and align the images onto a single plane. The implementation process is as follows: when a vehicle carrying an ID marker moves to a random position, the coordinates of the marker’s four corner points are detected. By comparing these coordinates with the actual side lengths of the marker, the initial homography matrix for rectification is calculated. Subsequently, several similar rectification iterations are performed on the rectified images to enhance accuracy. This process continues until the errors of the ID marker, measured at multiple random positions in the final rectified image, fall within the preset threshold range.

**Figure 4:**
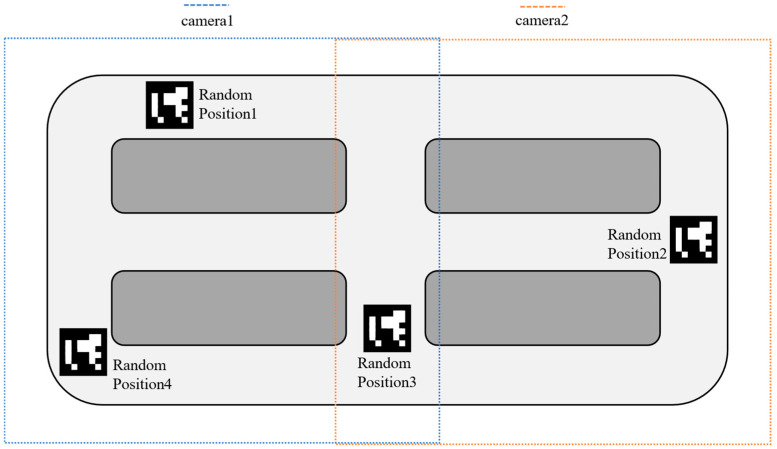
Recognition Results of the Dual-Camera System.

As illustrated in Figure 4, when the vehicle moves to non-overlapping areas such as Random Position 1, 2, and 4, the system continuously invokes the rectification algorithm to correct the images until the error falls below the preset threshold. When the vehicle enters the overlapping region of two cameras, such as Random Position 3, the system then calculates the cropping positions required for the subsequent image mosaicking algorithm.

The primary objective of this stage is to perform preprocessing computations and adaptive image calibration in response to significant disturbances encountered by the system. Since the design is tailored for fixed cameras and predefined stitching targets, preprocessing the data enables simplification of subsequent computational operations, thereby enhancing the overall algorithmic efficiency.

Using OpenCV’s getPerspectiveTransform() function, a linear perspective transformation matrix (M) is obtained. This matrix is then applied to the image data to perform perspective transformation, aligning each input frame with the unified reference coordinate system:

$$\left[\begin{array}{c} x^{\prime }\\ y^{\prime }\\ z^{\prime } \end{array}\right]=\left[\begin{array}{ccc} M_{11} & M_{12} & M_{13}\\ M_{21} & M_{22} & M_{23}\\ M_{31} & M_{32} & 1 \end{array}\right]\left[\begin{array}{c} x\\ y\\ 1 \end{array}\right]$$

Since the original image does not contain a *z*-axis dimension, its homogeneous coordinate is set to 1. However, after the perspective transformation, the corresponding value $z^{\prime }$ in the transformed image is no longer equal to 1, and thus normalization is required to obtain the final coordinates:

$$\left[\begin{array}{c} x^{\prime \prime }\\ y^{\prime \prime }\\ 1 \end{array}\right]=\frac{1}{z^{\prime }}\left[\begin{array}{c} x^{\prime }\\ y^{\prime }\\ z^{\prime } \end{array}\right]$$

Thus, we obtain the perspective transformation formula provided by OpenCV:

$$\mathrm{dst}\left(x,y\right)=\mathrm{src}\left(\frac{M_{11}x+M_{12}y+M_{13}}{M_{31}x+M_{32}y+M_{33}},\frac{M_{21}x+M_{22}y+M_{23}}{M_{31}x+M_{32}y+M_{33}}\right)$$

At this stage, we have obtained the perspective transformation formula provided by OpenCV. However, when using this transformation matrix directly, the resulting image only covers the region bounded by the four selected matching points. Therefore, it is necessary to adjust the canvas size and apply a further transformation to the matrix in order to obtain the complete transformed image.

As mentioned earlier, the transformation matrix obtained using the corresponding OpenCV function is given by:

$$M=\left[\begin{array}{ccc} M_{11} & M_{12} & M_{13}\\ M_{21} & M_{22} & M_{23}\\ M_{31} & M_{32} & 1 \end{array}\right]$$

We assume that the four corners of the original image ($\left(0,0\right),\left(0,w\right),\left(h,w\right),\left(h,0\right)$) are arranged clockwise starting from the top-left corner. By applying the perspective transformation to these four corner points, we can determine the boundary dimensions of the new canvas.

First, the coordinate points can be represented in matrix form as follows:

$$P=\left[\begin{array}{cccc} 0 & w & w & 0\\ 0 & 0 & h & h\\ 1 & 1 & 1 & 1 \end{array}\right]$$

By applying the perspective transformation matrix M to the coordinate matrix, we obtain $P^{\prime }=\mathrm{MP}$. Each element of $P^{\prime }$ is then divided by the last element in its corresponding column to perform normalization along the *z*-axis, thereby yielding the final corrected coordinates $P^{\prime \prime }$:

$$P^{\prime \prime }=\left[\begin{array}{cccc} P_{11} & P_{12} & P_{13} & P_{14}\\ P_{21} & P_{22} & P_{23} & P_{24}\\ 1 & 1 & 1 & 1 \end{array}\right]$$

The maximum and minimum values ($x_{\max},x_{\min}$)of the first row of the transformed coordinate matrix (corresponding to the *x*-axis) are obtained, as well as those ($y_{\max},y_{\min}$) of the second row (corresponding to the *y*-axis). The differences between these maximum and minimum values ($\Delta x=x_{\max}-x_{\min},\Delta y=y_{\max}-y_{\min}$) are then used to calculate the width and height of the new canvas.

To further translate the transformed image so that it fits entirely within the canvas, a translation operation is incorporated into the perspective transformation. Since the perspective transformation is performed by multiplying the coordinate matrix by M, this can be achieved by modifying M to include translation terms.

Create a unit matrix, and the negative values of the minimum x and y of $P^{\prime \prime }$ are placed in the third column of this matrix. This translation matrix is denoted as T.

$$T=\left[\begin{array}{ccc} 1 & 0 & -x_{\min}\\ 0 & 1 & -y_{\min}\\ 0 & 0 & 1 \end{array}\right]$$

Thus, the new transformation matrix is obtained as $M^{\prime }=\mathrm{TM}$. By multiplying $M^{\prime }$ with the coordinate matrix, the final transformed coordinates can be derived as follows:

$$\left[\begin{array}{c} x^{\prime \prime \prime }\\ y^{\prime \prime \prime }\\ 1 \end{array}\right]=\left[\begin{array}{c} x^{\prime \prime }-x_{\min}\\ y^{\prime \prime }-y_{\min}\\ 1 \end{array}\right]$$

The above derivation verifies the correctness of the proposed formulation; therefore, the final perspective transformation matrix is confirmed to be $M^{\prime }$.

Accordingly, the parameters for performing the image perspective transformation are given as follows:

$$\mathrm{warpPerspective}\left(\mathrm{src}=\mathrm{src},M=M^{\prime },\mathrm{dsize}=\left(\Delta x,\Delta y\right)\right)$$

Using the aforementioned function, the spatial coordinate systems of two images captured by the camera can be aligned into a common spatial coordinate system through a perspective transformation defined by the specified mapping coordinates.

#### 2.2.2. Image Stitching

In the first stage, since the images have already been geometrically corrected, it can be assumed that both image frames lie on the same physical plane. After applying a translation operation, the two images exhibit a high degree of overlap. Under these conditions, a combination of cropping and translational stitching can yield a final composite image that meets the requirements for target detection.

During the image stitching phase, when the vehicle carrying the identification marker enters the overlapping field of view of two cameras, efficient dual-camera image stitching can be achieved as follows: since multiple images have already been aligned into a common spatial coordinate system in prior steps, it suffices to translate the images along the x- and y-axes until the detected markers from both cameras coincide. Subsequently, a region around this overlapping area is selected, cropped, and seamlessly stitched to complete the process.

Considering that external disturbances and environmental changes during system operation may cause the preprocessed calibration data to become invalid, additional robustness mechanisms are introduced. Specifically, after the stitching is completed, the system continuously monitors the alignment and recognition status of the identification codes. If the stitched Aruco codes cannot be correctly detected during runtime, the system automatically recomputes the perspective transformation matrix and the cropping/stitching parameters to dynamically update the relevant configuration, ensuring consistent stitching performance.

Unlike conventional feature-based registration methods that rely on complex keypoint extraction and matching, the proposed approach employs a direct stitching strategy. This design substantially reduces computational complexity, making the method highly suitable for embedded implementation. Moreover, it significantly improves image processing efficiency, resulting in a stitching process with enhanced real-time performance and reduced latency, thereby satisfying the stringent real-time and low-latency requirements of engineering applications.

**Figure 5:**
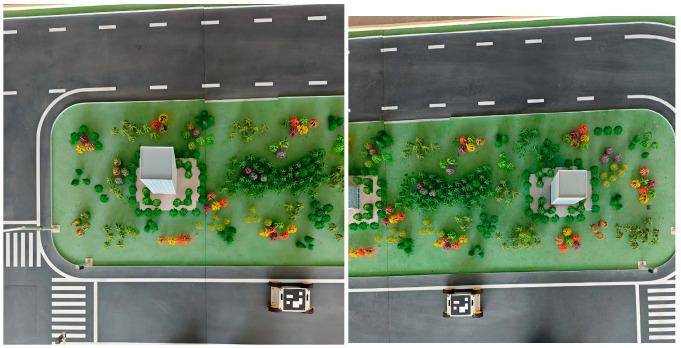
Raw Camera Data Input.

**Figure 6:**
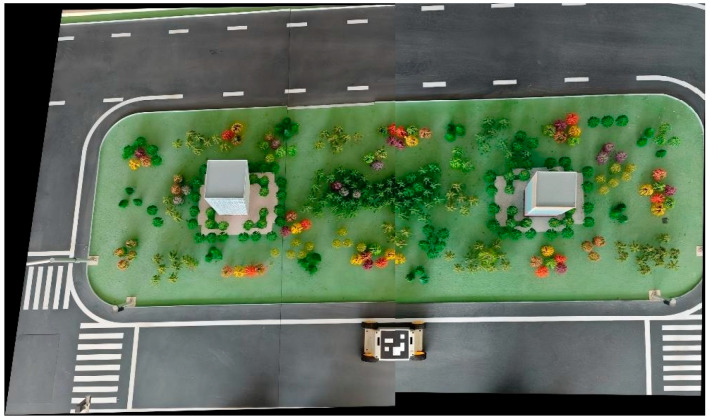
Stitched Video Output.

Figure 5 and Figure 6 illustrate the actual image mosaicking process. Figure 5 shows the raw data captured by the cameras. After rectification, a perspective transformation is applied to the entire canvas. Subsequently, the stitching algorithm identifies the center points of the markers present in both images, performs cropping at these locations, and then aligns and merges the images based on the marker positions.

## 3. Results

The video stitching method proposed in this paper is designed to meet the requirements of multi-camera image fusion in resource-constrained environments, emphasizing real-time performance and low-cost deployment rather than pursuing the high-precision fusion effects typical of conventional image stitching methods. Current mainstream video stitching techniques—such as feature-based panoramic stitching and multi-layer blending optimization—achieve superior visual quality but often demand significant hardware resources, exhibit high computational latency, and involve complex algorithmic designs, limiting their applicability in real-time engineering contexts.

To validate the feasibility and stability of the proposed method in vehicle identity code recognition scenarios, a series of experiments were conducted focusing on the impact of image stitching on target detection performance. By introducing representative perturbations, including variations in vehicle speed, driving angle, and identity code size, the experiments quantitatively evaluated whether the stitching boundary affects recognition performance. Additionally, the detection accuracy across the entire stitched image was analyzed to assess the overall performance and robustness of the proposed approach.

Prior to conducting the main experiments, we performed a comparative analysis with conventional algorithms, including SIFT, SURF, ORB, and optical flow LK. This comparison primarily focused on stitching speed. All experiments were conducted under the same local conditions. Similar to our proposed algorithm, these methods detected and identified Aruco markers in every frame to ensure the basic functions of the stitching algorithm were not compromised. The results are shown in Table 1:

Furthermore, we compared our method with CalibRBEV and UDIS regarding both stitching speed and recognition accuracy under the same conditions. The results are shown in Table 2:

The comparative experimental results indicate that, although the visual quality of the stitched images produced by our method is inferior to that of traditional methods when viewed by the human eye, our method achieves a leading position in terms of Aruco marker recognition effectiveness and stitching speed.

### 3.1. Experimental Design

The experimental evaluation focused on three key performance indicators:

(1) Detection rate, defined as the proportion of frames in which the identification marker is successfully detected by the system (number of frames with any identification marker detected/total number of frames analyzed).
(2) Recognition Accuracy, defined as the proportion of detected markers that match the ground-truth identity code (number of frames with correctly identified markers/number of frames with any identification marker detected).
(3) Stitching Accuracy—the positional accuracy of the detected identity code in the stitched image compared to its true reference position.

$$Stitching\;Accuracy=\frac{\frac{L_{x\_d}}{L_{x\_r}}+\frac{L_{y\_d}}{L_{y\_r}}}{2}$$

where $L_{x\_d}$ and $L_{y\_d}$ denote the projected distances along the x- and y-axes, respectively, from the detected coordinates of the identification marker to the reference coordinate; and $L_{x\_r}$ and $L_{y\_r}$ denote the projected distances along the x- and y-axes, respectively, from the ground-truth coordinates of the identification marker to the reference coordinate.

During the experiments, with respect to the first two metrics, the system attempted to recognize the vehicle’s identification marker in every frame, recording both the number of successful detections and the number of correctly identified instances. Each experimental condition was repeated 100 times, and the mean values were computed to ensure statistical validity.

The test experiment was conducted under a 1:50 scale scenario.

#### 3.1.1. Recognition Performance Under Different Vehicle Speeds

To evaluate the influence of the proposed stitching method on vehicle identity code recognition performance during actual operation, a speed variation experiment was conducted. In this test, scaled model vehicles were configured to traverse the overlapping region of two camera views at different speeds—10 cm/s, 15 cm/s, and 20 cm/s. Each vehicle was equipped with a standardized identity code label affixed to its roof. The recognition module utilized the Aruco marker detection algorithm provided by OpenCV for identity recognition. The results are shown in Table 3.

The experimental results indicate that when the vehicle speed is relatively low (below 15 cm/s), the system achieves superior recognition performance, with both the detection rate and recognition accuracy exceeding 96%. However, as vehicle speed increases, frame-to-frame discontinuities and motion blur effects become more pronounced. In particular, during frame fusion at the stitching boundary, parts of the identity code may fall along the edge of the blended region, affecting its visual integrity and consequently reducing recognition performance.

Although a noticeable decrease in recognition rate is observed at a speed of 20 cm/s, the recognition accuracy still remains above 81%, demonstrating that the proposed stitching method maintains strong recognition capability under moderate-speed conditions. These results confirm that the method can effectively meet the practical requirements of low-speed real-time recognition scenarios, making it well-suited for vehicle-in-the-loop semi-physical simulation systems.

#### 3.1.2. Recognition Performance Under Different Driving Angles

To further evaluate the stability and robustness of the proposed video stitching method under complex driving angles, an experiment was designed in which vehicles passed through the stitching region at varying approach angles. In real-world applications, vehicles may traverse the camera overlap region along non-perpendicular paths, leading to image distortion, edge occlusion, or stitching misalignment. Therefore, the impact of these angular variations on identity code recognition performance serves as a critical evaluation metric.

In this experiment, the model vehicle maintained a constant speed of 15 cm/s while entering the stitching region at different predefined approach angles of 0°, 15°, 30°, and 45°, corresponding to the angle between the vehicle’s trajectory and the principal viewing direction of the camera. All other experimental parameters were held constant to ensure controlled variable conditions. The results are shown in Table 4.

The experimental results demonstrate that when vehicles pass through the stitching region at small approach angles (≤15°), recognition performance remains consistently high. This indicates that the proposed stitching algorithm maintains excellent geometric consistency and image stability under minor angular deviations. However, as the approach angle increases—particularly at 30° or greater—image distortion within the stitching region becomes more pronounced. Portions of the identity code are either remapped or cropped, leading to a significant increase in detection failures and a consequent decline in overall recognition accuracy.

Although a decrease in recognition rate is observed at an angle of 45°, the method continues to achieve satisfactory performance for typical driving angles between 0° and 30°, demonstrating strong robustness in low-to-moderate angular conditions. Considering practical engineering requirements, this level of performance is sufficient to support low-speed, multi-directional recognition tasks in semi-physical vehicle simulation environments.

#### 3.1.3. Recognition Performance Under Different Identity Code Sizes

In video stitching and target recognition systems, target size has a direct influence on imaging quality and the precision of feature extraction. This effect is particularly critical in the stitching region, where image stretching, boundary cropping, and pixel interpolation can occur. Smaller targets are more susceptible to compression and blurring, thereby reducing the recognition algorithm’s reliability. To systematically evaluate the impact of identity code size on recognition performance within the stitching region, an identity code size variation experiment was conducted.

In this experiment, the vehicle maintained a constant speed of 15 cm/s, following a path perpendicular to the stitching boundary. Each vehicle carried an identity code placed centrally on its roof. Three physical code sizes were tested: 3 cm × 3 cm, 4 cm × 4 cm, and 6 cm × 6 cm. The structural design and pattern of the ArUco codes remained identical across tests, with only the physical dimensions varying. The results are shown in Table 5.

The experimental results show that the recognition performance improves significantly as the size of the identity code increases. When the code size is 3 cm × 3 cm, the number of pixels representing the code region becomes insufficient due to limited image resolution and stitching-induced distortions, making accurate detection and decoding difficult. Consequently, this configuration yields the lowest recognition rate. In contrast, the 4 cm × 4 cm identity code demonstrates considerably better recognizability, being successfully detected in most frames. The 6 cm × 6 cm code achieves near-optimal performance across all evaluation metrics, indicating that this size offers superior stability and resistance to stitching-induced perturbations under the current system configuration.

#### 3.1.4. Stitching Accuracy After Image Stitching

To verify the self-calibration capability of the proposed adaptive alignment algorithm, an experiment was conducted to assess both the stitching accuracy of repeated identity code localization and the resilience of the system under perturbation. The evaluation metric was defined as the difference between the detected identity code center position (measured from the top-left origin of the sandbox) and its corresponding true position, thereby quantifying the calibration precision.

In the experiment, the system’s accuracy was first measured during normal operation. Subsequently, disturbances were introduced by manually adjusting (or nudging) the cameras. The stitching accuracy was then recorded over several subsequent correction cycles to evaluate the system’s self-calibration capability. The results are shown in the Table 6.

The experimental results indicate that after the introduction of perturbations, the system was able to restore calibration effectively after a single calibration iteration. Following several subsequent iterations, the stitching accuracy stabilized at approximately 95%, demonstrating that the proposed system possesses strong positional recognition capability as well as robust self-calibration performance under external disturbances.

### 3.2. Experimental Result Analysis

Analysis of the experimental results reveals the following findings:

In the speed tests, the algorithm achieves a recognition rate above 93% when the scaled vehicle travels at speeds below 15 cm/s, which satisfies the requirements of most practical scenarios. However, at speeds exceeding 20 cm/s, the recognition rate declines due to frame misalignment in the stitching region and motion blur. This indicates that the current frame synchronization strategy requires further optimization for high-speed operation. One potential solution is to adaptively adjust illumination intensity and exposure time according to vehicle speed.

In the angular deviation tests, the identification marker maintains a recognition rate above 82% when the vehicle’s heading angle relative to the road direction is less than 30°. Beyond this threshold, excessive positional offset of the marker causes a sharp drop in recognition performance. Therefore, in real-world deployments, the vehicle’s trajectory should be constrained to maintain an angle of less than 30° relative to the road to ensure reliable system operation.

Regarding marker size, the 3 × 3 identification code achieves a recognition rate of 89%, which meets baseline requirements. Nevertheless, for enhanced robustness under practical conditions, larger marker sizes may be adopted where spatial constraints permit.

Furthermore, the system perturbation test demonstrated that when the entire system experiences substantial external disturbances, the integrated self-calibration module can promptly detect deviations and perform effective recalibration. This capability significantly enhances the system’s robustness and adaptability, ensuring stable performance even under environmental or mechanical variations.

## 4. Discussion

This study addresses the limited real-time performance commonly observed in conventional high-precision video stitching methods by proposing a fast video stitching algorithm optimized for fixed-scene applications. The developed system successfully stitches two 1440 × 960 video streams into a single 1440 × 1800 composite video at a stable frame rate of 30 frames per second.

Through a series of robustness experiments, the algorithm’s performance was systematically evaluated under three types of interference—vehicle speed variation, driving angle deviation, and identity code size change. Subsequently, external disturbances were simulated to test the system’s self-calibration capability, thereby providing practical validation and support for the applicability of the proposed algorithm. The results confirm that the proposed method maintains a high recognition success rate and strong resistance to disturbance, providing solid experimental support for its practical applicability.

Overall, the proposed video stitching approach achieves high detection reliability and robust performance in dynamic environments without relying on high-end hardware or complex geometric preprocessing. These characteristics verify the engineering feasibility and real-world value of the method, particularly for multi-vehicle cooperative semi-physical simulation systems and similar embedded vision applications.

## Figures and Tables

**Table 1:** Comparative Analysis of Frame Rates in Video Stitching Methods.

Method	SIFT	SURF	ORB	LK	Proposed Method
FPS	3	4	12	18	30

**Table 2:** Results of Bird’s-eye View Rectification Comparison.

Method	Detection Rate (%)	FPS
CalibRBEV	100	-
UDIS	88.24	25
Proposed Method	93.41	30

**Table 3:** Experimental Results under Different Vehicle Speeds.

Vehicle Speed (cm/s)	Detection Rate (%)	Recognition Accuracy (%)
10	96.48	99.225
15	93.41	99.08
20	81.93	98.75

**Table 4:** Experimental Results under Different Angles.

Vehicle Angle (°)	Detection Rate (%)	Recognition Accuracy (%)
0	93.41	99.08
15	82.7	99.79
30	82.6	98.1
45	65.72	97.38

**Table 5:** Experimental Results under Different-sized Identity Codes.

Marker Size (cm)	Detection Rate (%)	Recognition Accuracy (%)
3	89.03	98.23
4	93.41	99.08
6	98.26	99.16

**Table 6:** Results of Adaptive Correction Capability Test.

Initial Detection	Initial Disturbance	First Recalibration	Second Recalibration	Third Recalibration	Fourth Recalibration
94.86%	35.71%	92.86%	93.71%	95.14%	94.57%

## Data Availability

Data is contained within the article.
